# Supplementary material for: A Positive Causal Effect of Shrimp Allergy on Major Depressive Disorder Mediated by Allergy- and Immune-Related Pathways in the East Asian Population
Source: Nutrients. 2023 Dec 26;16(1):79. doi: 10.3390/nu16010079 (PMC10780813; doi:10.3390/nu16010079)
Supplement: Supplementary file 1 [file nutrients-16-00079-s001.zip › nutrients-2662599-supplementary.pdf]

Supplementary Table S1. Overall MR analyses with strong genetic instruments ($P<5\times10^{-6}$) for causal effects of SA on MDD.

Exposure	Outcome	N of SNPs in MRA [a]	MR p-value [b]	MR Method [c]	OR	95%LCI	95%UCI
SA	MDD	6	**1.76×10^{-2}**	Weighted median	1.07	1.01	1.14
			**5.01×10^{-3}**	Random-effects IVW	1.07	1.02	1.12
			**5.01×10^{-3}**	Fixed-effects IVW	1.07	1.02	1.12
			**8.50×10^{-3}**	RAPS	1.07	1.02	1.12

[a] N of SNPs in MRA: number of independent genetic SNPs used in the MR analysis for each pair of exposure and outcome; [b] MR p-value: p-value of the most suitable MR method; [c] MR Method: the most suitable MR analysis used in MR analysis. 95%LCI: 95% lower confidence interval; 95%UCI: 95% upper confidence interval; IVW, inverse-variance weighted; RAPS, Robust Adjusted Profile Score.
